# Elderly with knee osteoarthritis should perform nutritional assessment: integrative literature review

**DOI:** 10.1590/S1679-45082017RW3834

**Published:** 2017

**Authors:** Isabelle Ferreira da Silva Souza, Rosa Sá de Oliveira Neta, Juliana Maria Gazzola, Marcelo Cardoso de Souza

**Affiliations:** 1 Faculdade de Ciências da Saúde do Trairi, Universidade Federal do Rio Grande do Norte, Santa Cruz, RN, Brazil.; 2 Departamento de Fisioterapia, Universidade Federal do Rio Grande do Norte, Natal, RN, Brazil.

**Keywords:** Osteoarthritis, Knee, Nutrition assessment, Nutritional status, Aged

## Abstract

To review scientific literature to assess nutritional status of elderly patients with osteoarthritis in the last 16 years. This is an integrative literature review that included articles published in national and international journals indexed in PubMed, SciELO and BIREME. We selected 14 articles, and English language was predominant. The year of publication of articles ranged from 2006 to 2016, and most of papers were cross-sectional studies. To gather papers and for posterior evaluate, we used a validated data collection instrument and the included studies were critical analyzed by reading, gathering and analysis of articles. Studies suggested that there is a positive correlation between obesity and knee osteoarthritis. Obesity is one of the most important modifiable factors in worsening of osteoarthritis symptoms.

## RESUMO

O objetivo da pesquisa foi revisar a produção científica referente à avaliação do estado nutricional de idosos com osteoartrite nos últimos 16 anos. Assim, o estudo foi uma revisão integrativa da literatura, realizada com a busca de artigos publicados em periódicos nacionais e internacionais indexados no PubMed, na SciELO e na BIREME. Foram selecionados 14 artigos, e o idioma inglês foi preponderante. O período de publicação dos artigos variou de 2006 a 2016, com predominância de estudos do tipo transversais. Para reunir os artigos e para posterior avaliação, foi utilizado um instrumento de coleta de dados validado, e as análises críticas dos estudos incluídos foram realizadas por meio da leitura, do agrupamento e da análise dos artigos. As pesquisas sugeriram que existe correlação positiva entre obesidade e osteoartrite de joelhos. Além disto, a obesidade é um dos fatores mais significativos e modificáveis no agravamento dos sintomas da osteoartrite.

## INTRODUCTION

In the last decade, population aging and longevity of world population have progressed rapidly. According to statistical projections of the United Nations in Brazil, the world population of the elderly must reach 2 billion individuals by 2050.^1^

The Brazilian Institute of Geography and Statistics (IBGE - *Instituto Brasileiro de Geografia e Estatística*) reports that the population aged 60 and older represents a contingent of almost 15 million people, about 8.6% of the Brazilian population.^2^Therefore, projection s for 2025 is that Brazil will be at sixth position in relation to number of old people in the world.^3^

However, the association with changes because of aging, the elderly has more probability to be exposed to chronic diseases, such as osteoarthritis (OA). A study carried out with 1,769 individuals older than 60 years concluded that arthropathy is the main cause of physical disability among older people, and it is the second most frequent chronic condition found among this population, and this condition has a significant influence in functional dependency of these individuals.^4^

OA is chronic degenerative joint disease that cause joint, muscle, specific mechanical and biologic changes in which affect changes in cartilage of degradation and tissue associated with growth and cartilage remodeling. This disease affects directly daily life activities and, consequently, causes more vulnerability and dependency, therefore, contributing to reduce welfare and quality of life among elderlies.^5-7^

In addition, the two main changes found in body composition of elderly are reduction of muscle mass and accumulation of abdominal fat, which can cause non-transmissible chronic diseases and great impact in nutritional status of these individuals.^8^

Another important fact to consider is that obesity is of the main risk factors related to development of OA and this association of excessive body weight in development of OA exceeds the issue of mechanical overload. Obese elderlies with OA compared with non-obese elderlies present more risk to develop pain and any functional difficulties. There is evidence that this occur because obesity is directly related with inflammation, which contributing to worse knee OA.^9,10^

On the other hand, reduction of free fat muscle mass become evident after 60 years of age, mainly in changes in bone mineral density and amount of muscular mass, which cause reduction of muscle strength, difficult daily life activities, and, consequently, loss of independency of elderlies.^11^

Therefore, nutritional status assessment is needed among individuals with OA as well as monitoring of change in these body components, mainly the aging-related effects. For this reason, new intervention must be developed to achieve body weight control of older individuals and appropriate body composition for enabling health promotion, improvement of quality of life and reduction of OA impact in function of this population.

Considering the scarcity of current studies on the subject so far, new studies must be performed. This study reviews scientific output on nutritional status assessment of elderlies with OA in the last 16 years. We highlight the following guiding question of the study: from 2000 to 2016, what was the scientific output in nutritional assessment among elderlies with OA?

## METHODS

We searched for published articles in national and international journals indexed in the National Library of Medicine (PubMed), Scientific Electronic Library Online (SciELO) and Latin American and Caribbean Center on Health Sciences Information (BIREME). We used the keywords “osteoarthritis”, “aged”, “Nutrition Assessment” both in Portuguese and English.

Inclusion criteria for selection were: presence of chosen keywords in title and abstract, full text available on-line, studies written in Portuguese or English and published between January 2000 and July 2016.

We excluded descriptive studies without precisely information on method applied and/or results obtained, as well as meetings abstracts, incomplete or unavailable free full text articles, and papers not including keywords used in our search.

After databases consultation and search filtering, we identified and excluded other studies for duplicity. All abstract included were read. In case that abstract reading was not enough to establish if the article should or should not be include in the review, the full article was read to determine its eligibility for subsequent inclusion in the study. We included papers published between May and August 2016, resulting in 105 papers, however, in this review, we included 14 papers.

To collect papers and subsequent evaluate, we used a validated data collection instrument.^12^ Critical analysis of included studies were done by reading, grouping and analysis based on the instrument and inclusion and exclusion criteria. Findings are described in a table using a descriptive language, therefore, enable readers to identify applicability of results of this integrative review.

## RESULTS

**Figure 1:**
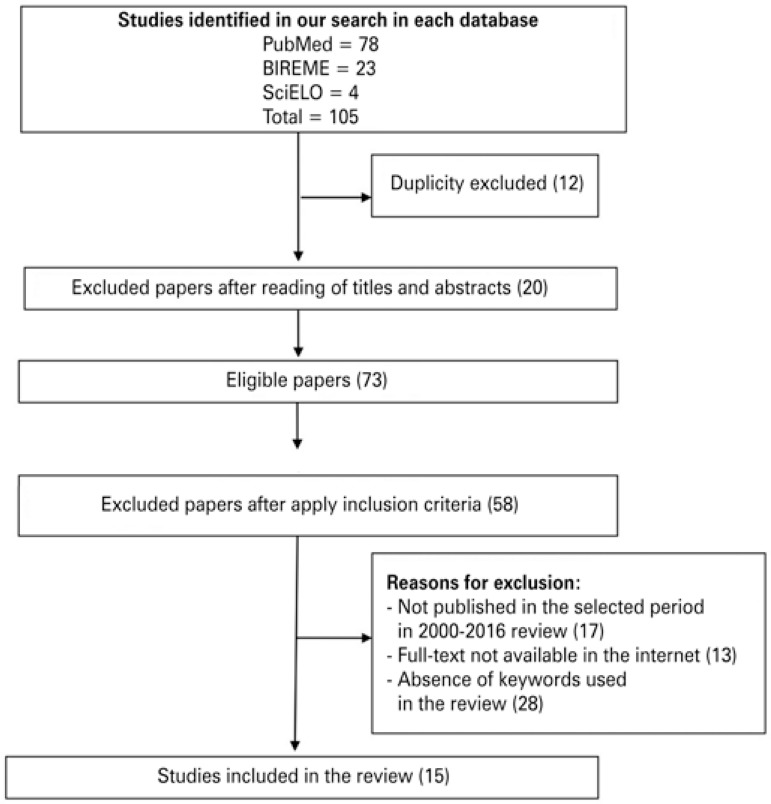
Flowchart of paper identification and selection for integrative review on osteoarthritis and nutritional assessment of elderlies between 2000 and 2016

Of 105 paper initially found in databases, 12 were excluded for duplicity, 20 were excluded after reading of titles and abstracts, and full text of 73 were read because they were compatible with guiding question and objective of the study. After critical analysis, we excluded 59 papers because did not meet inclusion criteria. The final sample was composed by 14 articles. A summary of papers selection process is shown in figure 1.

Of papers included 78 were found in PubMed (74%), 23 in BIREME (22%) and 4 in SciELO (4%). In terms of general characteristics of included papers, the oldest paper in our study collection was from 2006 and the newest from 2016. We observed a predominance of paper published in English language only, eight papers (53%) (Figure 1).

The analysis of characteristics of studies type revealed two prospective cohorts, one case-control study, and the remaining (12) were cross-sectional studies. Articles were published in 13 different journals: Revista Brasileira de Fisioterapia (one paper), Fisioterapia e Pesquisa (three papers), Fisioterapia em Movimento (one paper), Revista da Sociedade Brasileira de Clínica Médica (one paper), Europan Journal of Clinical Nutrition (one paper), Archives of Physical Medicine Rehabilitation (one paper), Arthritis Care (one paper), Arthritis Rheumatology (one paper), Aging and Disease (one paper), BMC Musculoskeletal Disorders (one paper), Yonsei Medical Journal (one paper); Rheumatology (one paper) and Calcified Tissue International (one paper).

Chart 1 describes papers according to authors, study type and results.

**Chart 1:** Abstracts on osteoarthritis and nutritional assessment of elderlies between 2000 and 2016

Author	Objective of the study	Type of the study	Results
Vasconcelos et al.^(13)^	To examine the influence of pain intensity, radiographic severity, obesity level and symptom duration on the functional capacity of obese individuals with knee OA	Cross-sectional	Pain intensity is a factor that influences functional activity performance among obese individuals with knee OA. In addition, clinically and radiographically, sample had moderate affection of knee OA
Vasconcelos et al.^(14)^	To compare the impact of the degree of obesity in symptoms and functional capacity of women with knee OA	Case-control	The degree of obesity had no impact on knee OA symptoms of pain, stiffness and functional difficulty in both obese women and women with morbid obesity
Chacur et al.^(15)^	To assess the correlations between body mass index, Waist circumference and Waist/Hip Circumference Ratio with Knee OA and observed association of these anthropometrical variables with severity of knee OA	Cross-sectional	Severity of OA was positive correlated with BMI and waist circumpherence
Rosis et al.^(16)^	To evaluate most and least affected joints, age patterns, sex, comorbidities and BMI, as well as its associations with appearance and development of OA in elderlies living long-term care institution	Cross-sectional	The high occurrence of OA in the population was related with advanced age, excessive weight and comorbidities that worsen the disease
Aurichio et al.^(17)^	To determine prevalence of obesity and its association in old population living in the city of São Carlos (SP, Brazil)	Cross-sectional	An association was found between the obese and presence of diabetes. In addition, women were more obese, reported joint pain and excessive body weight
Chacur et al.^(18)^	To determine possible correlations between anthropometric features, Q angle and knee OA in obese women	Cross-sectional	Abdominal obese, its degree and duration possibly contribute to incidence of knee OA in obese women
Christensen et al.^(19)^	To assess changes in micronutrient status (vitamin D, ferritin, and vitamin B12) and body composition in obese individuals after a dietary weight loss program	Prospective cohort	Weight loss can be successfully achieve in OA patients, especially if the diet includes enough nutrientes, therefore increasing bone mineral density and levels of vitamin D ande B12
Elbaz et al.^(20)^	To examine the associations of sex, body mass index, and age with knee OA symptomatic severity	Cross-sectional	Higher BMI correlated significantly with worse in knee OA symptoms
Qin et al.^(21)^	To examine the cross-sectional association between dietary magnesium intake and the radiographic knee OA among African-American and Caucasian men and women	Cross-sectional	Magnesium intake in the diet was inversely proportional to presence of knee OA in Caucasians but not in African Americans
Fahlman et al.^(22)^	To observe old individuals without OA at age 78, describing their height, weight and body mass index	Cross-sectional	Higher BMI is recognized as a risk factor for knee OA. Elderlies aged 78 years who were overweight did not have knee OA. A possible explanation may be an “inflated” BMI based on decrease in height, not just increase in weight
Holla et al.^(23)^	To assess whether BMI and depressed mood are independently associated with knee pain and activity limitations; and to compare the relative contributions of BMI and depressed mood to knee pain and activity limitations	Cross-sectional	In patients with knee OA, the BMI and depressed mood were positively and independently associated with knee pain and activity limitations
Lee et al.^(24)^	To examine the risk factors for OA and the contributing factors to current arthritic pain in older adults	Cross-sectional	Age, female gender, higher body mass index, and osteoporosis were significant risk factors for OA, while higher education level was a protective factor for OA
Weiss^(25)^	To determine whether BMI increases knee pain in individuals without severe knee OA	Cross-sectional	Weight loss may reduce knee OA pain even if the osteological symptoms are not treated
Ho-Phan et al.^(26)^	To investigate whether the association between BMI and OA is mediated by fat mass or lean mass	Cross-sectional	The association between body mass index and OA is mainly mediated by fat mass
Reyes et al.^(27)^	To analyze the effect of being overweight or obese on the incidence of routinely diagnosed knee, hip, and hand OA	Prospective cohort	Being overweight or obese increases the risk of hand, hip, and knee OA, with the greatest risk in the knee, and this occurs on response of increasing BMI

OA: osteoarthritis; BMI: body mass index.

Concerning to general features, oldest publication in our study collection was from 2006, of the 14 papers, 8 were published in international journals. Cross-sectional design was predominant and samples ranged between 28 and 4,769 individuals. All studies had different methods compared to number and sample composition, methods of body composition and anthropometrics. The results shown positive correlation of OA severity with excessive weight in almost all studies.^13-17,20,23-26^

## DISCUSSION

Osteoarthritis is progressive and irreversible disease in which a common finding is obesity. For this reason, to understand the impact of obesity in OA could provide information to health professionals and patients on how to reduce symptoms and complications of the disease.^28,29^

Lee et al., evaluated risk factors for OA in South Korean individuals and they found that advanced age (p=0.005), female gender (p<0.001), higher BMI (p<0.001) and osteoporosis (p<0.001) were significant associated factors with OA, while the higher education level (p=0.025) was a protective factor. In addition, higher BMI and unfavorable health status (p<0.001) were also considered significant factors that contributed to increase pain in OA among older population.^24^

One of hypothesis that possible explain the link between obesity and OA is the metabolic theory.^25,26^The reason is that pro-inflammatory factors mainly released by abdominal and visceral fat tissue, such as c-reactive protein (CRP), interleukin 6 (IL-6) and Plasminogen activator inhibitor type 1 (PAI-1), would adversely affect joint structure and accelerate development of OA.^13,30^

Biomechanical theory is broadly accepted. Vasconcelos et al.,^13^ cited functional difficulties of obese in locomotion activities that require movement and weight bearing on joints. This theory is corroborated in the literature,^31-33^ and it indicates that displacement of gravity center because of presence of abdominal protrusion, lead to pelvic anteversion, knee valgus and flat feet that is associated with excessive weight and changes in gait, which cause joint stress.^13^

Findings of this review showed that severity of OA correlated positively with excessive weight and distribution of body fat in almost all studies.^13,14,16,18,20,22-26^ The majority of studies that proposed to evaluate nutritional status of elderlies used as reference parameters the body mass index (BMI), but the study by Chacur et al., was the only one to consider also abdominal fat by the measurement of waist circumference (WC).^15,18^

Fahlman et al.,^22^drawn the attention to the fact that higher BMI is not reliable indicator only for weight gain, but it also reliable for reduce height because of age and reduce of bone mass.

Ho-phan et al., were beyond anthropometrical measures and considered only measures of body composition obtained from Dual-energy X-ray absorptiometry (DXA) that measured lean mass, fat mass and bone densitometry. We believe that BMI was broadly used in these studies because it has more practical value, and it also the easiest and economic manner than DXA to diagnosed obesity.^26^

It is also important to remember that BMI represents only a change in energetic balance, therefore unable the analysis of metabolic changes or composition of lean mass or fat mass. In addition, it is possible that these factors not covered by BMI would have greater repercussion in functional capacity of obese individuals.^13^

Rosis et al., found a prevalence of approximately 52% among older individuals with OA who were classified as obese and overweight,^16^ this classification would be related to worsen of symptoms. However, Aurichio et al., did not use in its method specific issues related to OA, but about symptoms related to rheumatism and rheumatoid arthritis but it was not sensitive to cases of OA. However, perhaps the association between joint pain and obesity could be explained by presence of OA in these older individuals, and this would agree with finding in the literature.^17^

Chacur et al., showed in their study that women with BMI>34kg/m^2^ and WC>110cm had 3.7 and 7 times, respectively, more change of presenting OA.^15^

However, Vasconcelos et al., showed that degree of obesity did not impact pain symptoms, stiffness and functional difficulties associated with knee OA in women in different obesity degrees (obese and morbid obese) because both had similar performance, suggesting that other factors would influence functional performance of obese with knee OA.^14^

Based on this fact, there is need to promote weight control as way to improve symptoms of OA. Christensen et al.,^19^ suggested the use of low energy consumption diet, since it contains an enough amount of nutrients. In their study, these authors promoted weight loss, increase of bone mineral density and improvement of levels of vitamin D and B12 in a safe format with therapy of weight loss induced by diet in obese patients with OA.

The study by Qin et al., was single one to determine food intake and to confirm an inversely proportional association between magnesium intake in diet and knee OA in white individuals. The same result was not found among Afro-American, therefore, suggesting the need of further studies to clarify possible action mechanisms in terms of race.^21^ Holla et al., also innovated in their study by determine association among BMI, depressed mood and pain limitations in the knee because of OA, however, they found that this association was positive and independent, and this contribution to BMI to physical limitation seem to be more substantial than depressed mood.^23^

Ho-Phan et al.,^26^also had differential findings of several studies in this review when they evaluated nutritional status of asymptomatic individuals with OA. These researchers verified that, in men, nor fat mass neither lean mass was associated with knee OA. On the other hand, in women the risk for knee OA was significantly associated with higher fat mass and not with lean mass. This finding is justified because the higher the body fat the greater the increase in strength and weight bearing on joints, and it can also cause degradation in cartilage and osteoarthritis.^31^

A prospective cohort study by Reyes et al., developed for 4.45 years with BMI data of subjects aged ≥40 years who did not present OA found that those with excessive weight or obesity had increased risk to develop knees, hip and hands OA, but especially in knees compared with individuals with normal weight. The excessive weight and degree I and II obesity increase the risk of OA in knee by 2 times, 3.1 times, and 4.7 times factor, respectively, in their study.^27^

All studies discussed showed a positive correlation between overweight/obesity and OA, therefore, a relationship may exist between weight loss and pain improvement. Messier et al.,^33^ observed benefits in diet and exercise of overweight or obese individuals with knee OA. They observed that after 18 weeks the mean of weight loss was higher in group that combined diet and exercise than in the group that only adopted diet, and this latter had better result than those who only exercised. In addition, the diet and exercise group had less inflammation, less pain, improvement in functionality, faster gait, and improvement in quality of life.^5^

Our review shows the importance to diagnose OA and its associations, highlighting the need to develop epidemiologic studies including different population, mainly because OA has a considerable differences in clinical presentation based on sex, age range, and BMI of individuals, therefore turning description of these variables relevant for assessment of specific population.

Further studies should be done with older individuals with OA because studies included In our study, in addition to use similar methods, did not present multidimensional assessment among older people. In addition, there is the need of adequate nutritional diagnosis for those affected by the disease to establish efficacy interventions in weight control, and, consequently, reduce symptoms of OA to promote better quality of live among older individuals with OA.

## CONCLUSION

Studies suggest a positive correlation between obesity and development of knee OA. Still, obesity is one of the most significant changeable factors in worsening of osteoarthritis symptoms.
